# Supplementary figures and images for: Chikungunya virus in Europe: A retrospective epidemiology study from 2007 to 2023
Source: PLoS Negl Trop Dis. 2025 Mar 7;19(3):e0012904. doi: 10.1371/journal.pntd.0012904 (PMC11906167; doi:10.1371/journal.pntd.0012904)

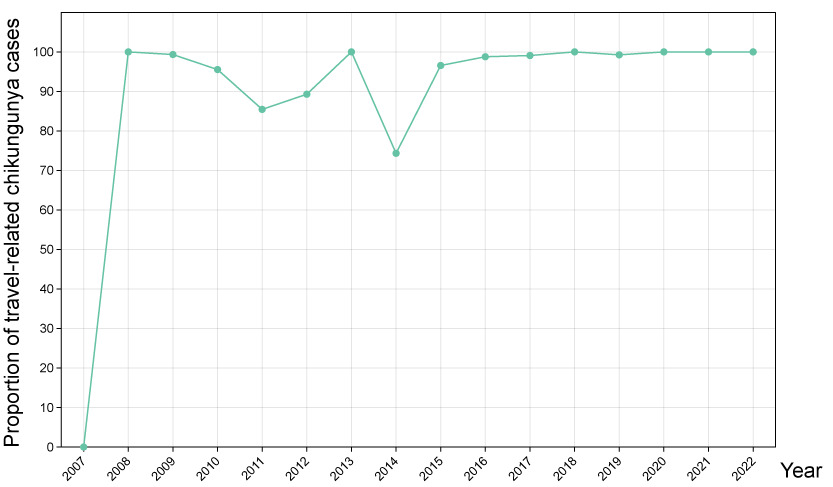

Supplement: S1 Fig — Line plot showing the proportion of travel-related chikungunya cases recorded each year in Europe. (TIF) [file pntd.0012904.s001.tif]

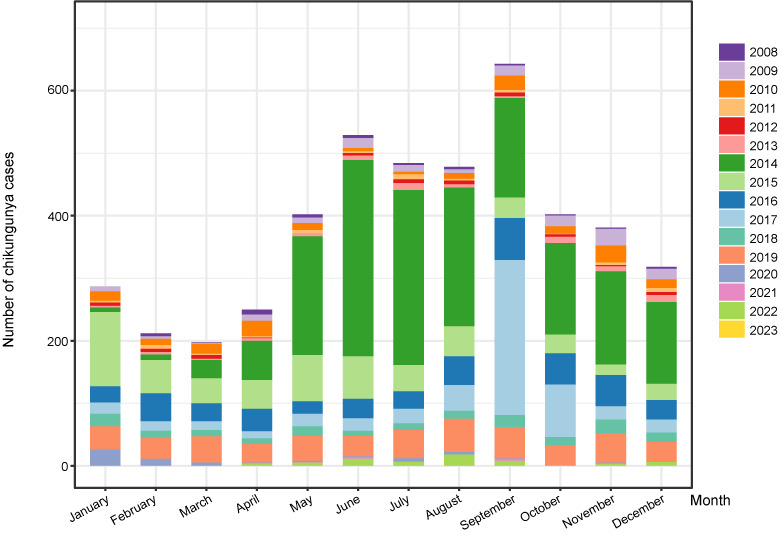

Supplement: S2 Fig — Number of chikungunya cases per month during 2008–2023 in Europe. (TIF) [file pntd.0012904.s002.tif]

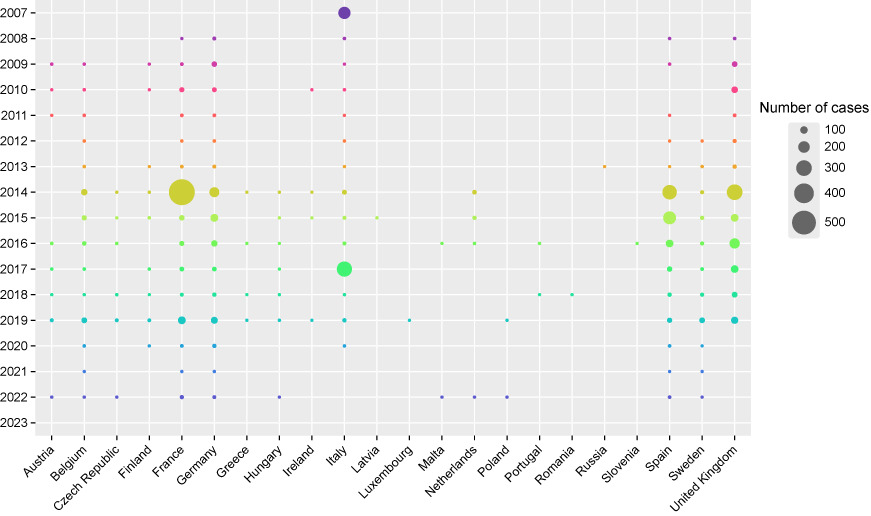

Supplement: S3 Fig — (TIF) [file pntd.0012904.s003.tif]
