# Supplementary material for: Chikungunya virus in Europe: A retrospective epidemiology study from 2007 to 2023
Source: PLoS Negl Trop Dis. 2025 Mar 7;19(3):e0012904. doi: 10.1371/journal.pntd.0012904 (PMC11906167; doi:10.1371/journal.pntd.0012904)
Supplement: S1 Table — (DOCX) [file pntd.0012904.s004.docx]

**S1** **Table** Number of chikungunya cases reported for each country per year between 2007 and 2023.

| **Year** | **2007** | **2008** | **2009** | **2010** | **2011** | **2012** | **2013** | **2014** | **2015** | **2016** | **2017** | **2018** | **2019** | **2020** | **2021** | **2022** | **2023** |
| --- | --- | --- | --- | --- | --- | --- | --- | --- | --- | --- | --- | --- | --- | --- | --- | --- | --- |
| Italy | 217 | 9 | 3 | 7 | 2 | 5 | 3 | 39 | 18 | 17 | 289 | 4 | 25 | 6 | 0 | 0 | 0 |
| France | 0 | 1 | 13 | 44 | 12 | 6 | 11 | 550 | 52 | 42 | 35 | 16 | 108 | 13 | 3 | 23 | 0 |
| Spain | 0 | 5 | 6 | 0 | 4 | 2 | 2 | 272 | 234 | 105 | 51 | 27 | 46 | 9 | 1 | 10 | 0 |
| United Kingdom | 0 | 9 | 56 | 79 | 14 | 21 | 26 | 301 | 106 | 169 | 104 | 59 | 94 | 0 | 0 | 0 | 0 |
| Germany | 0 | 17 | 54 | 37 | 13 | 9 | 16 | 162 | 110 | 74 | 33 | 26 | 87 | 26 | 4 | 16 | 0 |
| Belgium | 0 | 0 | 6 | 8 | 8 | 6 | 7 | 74 | 44 | 29 | 10 | 3 | 60 | 8 | 2 | 3 | 0 |
| Sweden | 0 | 0 | 0 | 0 | 0 | 2 | 6 | 19 | 23 | 20 | 13 | 20 | 58 | 1 | 2 | 1 | 0 |
| Netherlands | 0 | 0 | 0 | 0 | 0 | 0 | 0 | 33 | 24 | 7 | 0 | 0 | 0 | 0 | 0 | 1 | 0 |
| Austria | 0 | 0 | 8 | 2 | 2 | 0 | 0 | 0 | 0 | 9 | 5 | 1 | 17 | 0 | 0 | 3 | 0 |
| Czech Republic | 0 | 0 | 0 | 0 | 0 | 0 | 0 | 3 | 1 | 7 | 0 | 6 | 15 | 0 | 0 | 2 | 0 |
| Finland | 0 | 0 | 3 | 1 | 0 | 0 | 1 | 4 | 7 | 0 | 5 | 1 | 14 | 2 | 0 | 0 | 0 |
| Hungary | 0 | 0 | 0 | 0 | 0 | 0 | 0 | 2 | 2 | 1 | 1 | 3 | 5 | 0 | 0 | 2 | 0 |
| Greece | 0 | 0 | 0 | 0 | 0 | 0 | 0 | 1 | 0 | 2 | 0 | 1 | 2 | 0 | 0 | 0 | 0 |
| Portugal | 0 | 0 | 0 | 0 | 0 | 0 | 0 | 0 | 0 | 3 | 0 | 1 | 0 | 0 | 0 | 0 | 0 |
| Ireland | 0 | 0 | 0 | 1 | 0 | 0 | 0 | 1 | 1 | 0 | 0 | 0 | 1 | 0 | 0 | 0 | 0 |
| Latvia | 0 | 0 | 0 | 0 | 0 | 0 | 0 | 0 | 2 | 0 | 0 | 0 | 0 | 0 | 0 | 0 | 0 |
| Poland | 0 | 0 | 0 | 0 | 0 | 0 | 0 | 0 | 0 | 0 | 0 | 0 | 2 | 0 | 0 | 2 | 0 |
| Romania | 0 | 0 | 0 | 0 | 0 | 0 | 0 | 0 | 0 | 0 | 0 | 2 | 0 | 0 | 0 | 0 | 0 |
| Slovenia | 0 | 0 | 0 | 0 | 0 | 0 | 0 | 0 | 0 | 2 | 0 | 0 | 0 | 0 | 0 | 0 | 0 |
| Luxembourg | 0 | 0 | 0 | 0 | 0 | 0 | 0 | 0 | 0 | 0 | 0 | 0 | 1 | 0 | 0 | 0 | 0 |
| Malta | 0 | 0 | 0 | 0 | 0 | 0 | 0 | 0 | 0 | 1 | 0 | 0 | 0 | 0 | 0 | 1 | 0 |
| Russia | 0 | 0 | 0 | 0 | 0 | 0 | 1 | 0 | 0 | 0 | 0 | 0 | 0 | 0 | 0 | 0 | 0 |
