# Supplementary material for: Chikungunya virus in Europe: A retrospective epidemiology study from 2007 to 2023
Source: PLoS Negl Trop Dis. 2025 Mar 7;19(3):e0012904. doi: 10.1371/journal.pntd.0012904 (PMC11906167; doi:10.1371/journal.pntd.0012904)
Supplement: S2 Table — (DOCX) [file pntd.0012904.s005.docx]

**S2** **Table** Comparison of the number of chikungunya cases by regions of Europe

|  | **2008-2010** | **2011-2013** | **2014-2016** | **2017-2019** | **2020-2022** |
| --- | --- | --- | --- | --- | --- |
| **Northern Europe** | 5 (1.4%) | 9 (5.0%) | 75 (2.9%) | 112 (9.0%) | 6 (4.3%) |
| **Eastern Europe** | 0 | 1 (0.6%) | 2 (0.1%) | 0 | 0 |
| **Southern Europe** | 30 (1.4%) | 18 (10.1%) | 693 (26.9%) | 448 (35.8%) | 26 (18.6%) |
| **Western Europe** | 216 (58.5%) | 111 (62.0%) | 1431 (55.6%) | 490 (39.2%) | 53 (37.9%) |
| **Central Europe** | 118 (32.0%) | 40 (22.3%) | 371 (14.4%) | 201 (16.1%) | 55 (9.3%) |
| **Total** | 369 | 179 | 2572 | 1251 | 140 |
