# Supplementary material for: Chikungunya virus in Europe: A retrospective epidemiology study from 2007 to 2023
Source: PLoS Negl Trop Dis. 2025 Mar 7;19(3):e0012904. doi: 10.1371/journal.pntd.0012904 (PMC11906167; doi:10.1371/journal.pntd.0012904)
Supplement: S3 Table — (DOCX) [file pntd.0012904.s006.docx]

**S3** **Table** Age-standardized rate of chikungunya cases reported for each country per year between 2008 and 2023.

| **Year** | **2008** | **2009** | **2010** | **2011** | **2012** | **2013** | **2014** | **2015** | **2016** | **2017** | **2018** | **2019** | **2020** | **2021** | **2022** |
| --- | --- | --- | --- | --- | --- | --- | --- | --- | --- | --- | --- | --- | --- | --- | --- |
| Austria | 0.00 | 9.50 | 2.45 | 2.20 | 0.00 | 0.00 | 0.00 | 0.00 | 10.30 | 5.75 | 0.99 | 18.54 | 0.00 | 0.00 | 3.93 |
| Belgium | 0.00 | - | - | - | - | 6.64 | 68.59 | 40.98 | 26.50 | 8.65 | 2.97 | 53.05 | 6.70 | 1.95 | 2.75 |
| Czechia | 0.00 | 0.00 | 0.00 | 0.00 | 0.00 | 0.00 | 2.91 | 0.91 | 7.01 | 0.00 | 5.87 | 14.87 | 0.00 | 0.00 | 2.18 |
| Finland | 0.00 | 6.49 | 2.17 | 0.00 | 0.00 | 2.18 | 8.20 | 13.75 | 0.00 | 9.74 | 1.76 | 25.81 | 4.14 | 0.00 | 0.00 |
| France | 0.15 | 0.50 | 6.84 | 1.89 | 1.80 | 1.92 | 54.98 | 10.94 | 6.62 | 5.45 | 2.34 | 16.66 | 1.96 | 0.43 | 3.56 |
| Germany | 1.98 | 6.56 | 4.54 | 1.57 | 1.13 | 1.99 | 0.95 | 14.53 | 9.25 | 4.32 | 3.30 | 10.51 | 3.14 | 0.45 | 2.01 |
| Greece | 0.00 | 0.00 | 0.00 | 0.00 | 0.00 | 0.00 | 0.95 | 0.00 | 2.01 | 0.00 | 1.04 | 2.13 | 0.00 | 0.00 | 0.00 |
| Hungary | 0.00 | 0.00 | 0.00 | 0.00 | 0.00 | 0.00 | 2.04 | 2.14 | 1.04 | 0.95 | 2.95 | 5.33 | 0.00 | 0.00 | 2.24 |
| Ireland | 0.00 | 0.00 | 2.03 | 0.00 | 0.00 | 0.00 | 2.08 | 2.29 | 0.00 | 0.00 | 0.00 | 2.10 | 0.00 | 0.00 | 0.00 |
| Italy | 1.52 | 2.08 | 1.28 | 0.35 | 0.96 | 0.54 | 6.07 | 3.15 | 2.63 | 43.71 | 0.64 | 4.03 | 1.02 | 0.00 | 0.00 |
| Latvia | 0.00 | 0.00 | 0.00 | 0.00 | 0.00 | 0.00 | 0.00 | 10.70 | 0.00 | 0.00 | 0.00 | 0.00 | 0.00 | 0.00 | 0.00 |
| Luxembourg | 0.00 | 0.00 | 0.00 | 0.00 | 0.00 | 0.00 | 0.00 | 0.00 | 0.00 | 0.00 | 0.00 | 15.55 | 0.00 | 0.00 | 0.00 |
| Malta | 0.00 | 0.00 | 0.00 | 0.00 | 0.00 | 0.00 | 0.00 | 0.00 | 21.98 | 0.00 | 0.00 | 0.00 | 0.00 | 0.00 | 16.57 |
| Netherlands | 0.00 | 0.00 | 0.00 | 0.00 | 0.00 | 0.00 | - | - | - | 0.00 | 0.00 | 0.00 | 0.00 | 0.00 | - |
| Poland | 0.00 | 0.00 | 0.00 | 0.00 | 0.00 | 0.00 | 0.00 | 0.00 | 0.00 | 0.00 | 0.00 | 0.54 | 0.00 | 0.00 | 0.49 |
| Portugal | 0.00 | 0.00 | 0.00 | 0.00 | 0.00 | 0.00 | 0.00 | 0.00 | 2.89 | 0.00 | 0.75 | 0.00 | 0.00 | 0.00 | 0.00 |
| Romania | 0.00 | 0.00 | 0.00 | 0.00 | 0.00 | 0.00 | 0.00 | 0.00 | 0.00 | 0.00 | 1.06 | 0.00 | 0.00 | 0.00 | 0.00 |
| Slovenia | 0.00 | 0.00 | 0.00 | 0.00 | 0.00 | 0.00 | 0.00 | 0.00 | 10.08 | 0.00 | 0.00 | 0.00 | 0.00 | 0.00 | 0.00 |
| Spain | - | - | 0.00 | - | - | - | 56.75 | 45.29 | 22.25 | 11.51 | 6.19 | 9.44 | 2.01 | 0.24 | 2.06 |
| Sweden | 0.00 | 0.00 | 0.00 | 0.00 | 2.39 | 5.70 | 21.12 | 22.70 | 21.42 | 12.25 | 19.70 | 57.79 | 0.80 | 2.13 | 0.99 |
| United Kingdom | 1.50 | 9.04 | 12.82 | 2.28 | 3.47 | 4.17 | 47.26 | 16.42 | 26.46 | 16.18 | 9.17 | 14.80 | 0.00 | 0.00 | 0.00 |

-: data unavailable.
