# Supplementary material for: Chikungunya virus in Europe: A retrospective epidemiology study from 2007 to 2023
Source: PLoS Negl Trop Dis. 2025 Mar 7;19(3):e0012904. doi: 10.1371/journal.pntd.0012904 (PMC11906167; doi:10.1371/journal.pntd.0012904)
Supplement: S4 Table — (DOCX) [file pntd.0012904.s007.docx]

**S4** **Table** Number of cases per country per month in 2014.

| Month/Country | Jan | Feb | Mar | Apr | May | Jun | Jul | Aug | Sept | Oct | Nov | Dec |
| --- | --- | --- | --- | --- | --- | --- | --- | --- | --- | --- | --- | --- |
| Italy | 0 | 0 | 0 | 0 | 0 | 0 | 1 | 1 | 35 | 2 | 0 | 0 |
| France | 0 | 4 | 18 | 28 | 105 | 160 | 120 | 98 | 14 | 3 | 0 | 0 |
| Spain | 0 | 2 | 3 | 30 | 65 | 82 | 75 | 14 | 1 | 0 | 0 | 0 |
| United Kingdom | 2 | 3 | 8 | 3 | 5 | 9 | 11 | 20 | 52 | 67 | 71 | 60 |
| Germany | 0 | 0 | 0 | 0 | 0 | 0 | 0 | 0 | 17 | 30 | 65 | 50 |
| Belgium | 0 | 0 | 0 | 0 | 5 | 16 | 23 | 16 | 14 | 0 | 0 | 0 |
| Sweden | 0 | 0 | 0 | 1 | 0 | 5 | 7 | 6 | 0 | 0 | 0 | 0 |
| Netherlands | 0 | 0 | 0 | 1 | 6 | 7 | 8 | 9 | 2 | 0 | 0 | 0 |
| Czech Republic | 0 | 0 | 0 | 0 | 0 | 1 | 1 | 1 | 0 | 0 | 0 | 0 |
| Finland | 0 | 0 | 0 | 0 | 1 | 1 | 1 | 1 | 0 | 0 | 0 | 0 |
| Hungary | 0 | 0 | 0 | 0 | 0 | 1 | 0 | 1 | 0 | 0 | 0 | 0 |
| Greece | 0 | 0 | 0 | 0 | 0 | 0 | 1 | 0 | 0 | 0 | 0 | 0 |
| Ireland | 0 | 0 | 0 | 0 | 0 | 1 | 0 | 0 | 0 | 0 | 0 | 0 |
