# Supplementary material for: Chikungunya virus in Europe: A retrospective epidemiology study from 2007 to 2023
Source: PLoS Negl Trop Dis. 2025 Mar 7;19(3):e0012904. doi: 10.1371/journal.pntd.0012904 (PMC11906167; doi:10.1371/journal.pntd.0012904)
Supplement: Attachment — Submitted filename: PNTD review.pdf [file pntd.0012904.s008.pdf]

Review Comments:

The presented work was a pleasure to read. I would like to thank the author's for submitting a very polished and complete manuscript for review. The work provides valuable insight following the careful review and statistical and comparative analyses of available data from 2007-2023. I am pleased to announce that I could only find three areas for your review/correction:

36 Guadeloupe8.7%) = please add space between

56 Guadeloupe8.7%), = please add space between

141 Europe has traditionally been categorized into 142 five regions, including Central, Eastern, Southern, Western and Northern Europe. = This could use a reference to define these boundaries. Perhaps a bit petty, but I think it helps to ensure the epidemiological analysis is clearly defined by solid reference for these exact boundaries.
