# Supplementary material for: Chikungunya virus in Europe: A retrospective epidemiology study from 2007 to 2023
Source: PLoS Negl Trop Dis. 2025 Mar 7;19(3):e0012904. doi: 10.1371/journal.pntd.0012904 (PMC11906167; doi:10.1371/journal.pntd.0012904)
Supplement: Attachment — Submitted filename: Response-R1.docx [file pntd.0012904.s009.docx]

Dear Editor,

Thank you for your decision letter concerning our manuscript (PNTD-D-24-01472) entitled "Chikungunya virus in Europe: a retrospective epidemiology study from 2007 to 2023", and your time regarding for our revision. I also appreciate all the critical comments from reviewers. We have carefully considered the comments and revised the manuscript accordingly. With these improvements, we hope that the current version can meet the Journal’s standards for publication. The following is a point-by-point response to all those comments and a list of changes we have made to the manuscript.

Reviewers' comments:

**Reviewer** **#1:**

The results of the analyses provide an epidemiological review of the target organism within the defined timeline. The data are presented well and with clarity. I have no concerns.

Response: We would like to thank the respected reviewer #1 for all those useful comments. According to your suggestion, we have revised our manuscript accordingly.

Q1. 36 Guadeloupe8.7%) = please add space between

R1: We have added it now (line 36).

Q2. 56 Guadeloupe8.7%), = please add space between

R1: We have added it now (line 57).

Q3. 141 Europe has traditionally been categorized into 142 five regions, including Central, Eastern, Southern, Western and Northern Europe. = This could use a reference to define these boundaries. Perhaps a bit petty, but I think it helps to ensure the epidemiological analysis is clearly defined by solid reference for these exact boundaries.

R1: Yes, we have added a reference now (line 144).

**Reviewer #2:**

Q1. The analysis presented coincides with the plan.

Line 203 to 206. I think this information would look better in a table distributing the countries and months of specific peaks.

R1. We would like to thank the respected reviewer #2 for the useful comment. We have added it now. Please see the line 206 and Table S4.

Q2. Undoubtedly, the increase in travel and the transit of goods and people can be a risk factor to take into account, but it is not the only one. Climate change is positioning itself as a more important base factor. For this reason, I do not think it is appropriate to justify the reduction of travel to prevent the transmission of CHIKV.

R2. Thanks for the good suggestions. We agree with the reviewer. We have revised the relevant content in the article with followings: “With the absence of specific antiviral treatments and vaccines still in development, surveillance and vector control are essential in suppressing the re-emergence and epidemics of CHIKV”, and “the most effective tools to combat the disease in Europe involve surveillance and vector control”.

Please see lines 42-44 and 318-319.

Q3. Line 282 to 283. This statement should be supported by entomological data on the infestation of both vectors over the years monitored. If there is no information on the matter, it could not be affirmed, the infestation by both vectors collaborates with the increase in transmission.

R3. We agree with the reviewer. We have deleted this sentence here.

Q4. I believe that the definitions expressed here should be clarified and separated. There should be a definition of probable case and one for confirmed case. Also for any of them add the date of return to the country.

R4. We have added the definition of probable case and one for confirmed case with following (lines 105-110). “A case must meet at least one of the following four criteria to be considered a confirmed case: CHIKV isolated from a clinical specimen, detection of virus nucleic acid, chikungunya specific IgM antibodies were detected and confirmed by neutralization, seroconversion or four-fold antibody titer increase of chikungunya specific antibodies. Probable cases were defined as the presence of chikungunya specific IgM antibodies in a single serum sample”. The criteria for these definitions come from the European Centre for Disease Prevention and Control (https://www.ecdc.europa.eu/en/all-topics/eu-case-definitions). However, the date of return to the country were unavailable from the European Centre for Disease Prevention and Control. Thus, we did not add the relevant information. We are sorry for that.

Q5. Line 187 to 190. These data are not clear from what was said in the previous sentence, where it is said that most of them were acquired in North America.

R5. Thanks for the comment.

Among the 2836 travel-related cases, the data regarding the probable country of infection for 963 cases was unavailable. The remaining 1873 cases had available information on the probable country of infection, with the majority acquired in North America (n=835), followed by Asia (n=672) and South America (n=316).

A total of seven countries from Aisa: India (n=332), Thailand (n=222), Myanmar (n=41), Indonesia (n=30), Bangladesh (n=28), Philippines (n=18), and Malaysia (n=1).

A total of five countries from North America: Dominican Republic (n=256), Guadeloupe (n=247), Martinique (n=170), Jamaica (n=105), and Haiti (n=57).

Therefore, the probable countries with the highest number of returned infections were India (332, 11.7%), followed by Dominican Republic (256, 9.0%), Guadeloupe (247, 8.7%), Thailand (222, 7.8%), and Martinique (170, 6.0%).

Please also see the figure 3 and lines 185-188.
